# Supplementary material for: Unveiling Coverage-Dependent Interactions of N-Methylaniline with the Pt(111) Surface
Source: J Phys Chem C Nanomater Interfaces. 2025 Mar 21;129(13):6196–210. doi: 10.1021/acs.jpcc.4c08116 (PMC11973917; doi:10.1021/acs.jpcc.4c08116)
Supplement: Supplementary file 1 — jp4c08116_si_001.pdf [file jp4c08116_si_001.pdf]

Supporting Information

Unveiling Coverage-Dependent Interactions of N-Methylaniline with the Pt(111) Surface

Bushra Ashraf[‡a], *Nils Brinkmann*[‡ b], *Dave Austin*[a], *Duy Le*[a], *Katharina Al-Shamery*[b*], *Talat S. Rahman*[a*]

[a]Department of Physics, University of Central Florida, Orlando, FL 32816, USA

[b]Institute of Chemistry, Carl von Ossietzky University of Oldenburg, Carl-von-Ossietzky-Straße 9-11, 26129 Oldenburg, Germany

[*]Email: Talat S. Rahman (talat@ucf.edu);

Katharina Al-Shamery (katharina.al.shamery@uni-oldenburg.de)

Table of Contents

1. The band structure of bulk Pt……………………………………………………………………………..1

2. The Pt(111) surface…………………………………………………………………………………….....1

3. Projected density of states for the Pt(111) surface and the NMA molecule……………………………………..2

4. Determination of the adsorption site of NMA on the Pt(111) surface…………………………………………..3

5. Binding of the benzene ring and ammonia to Pt(111)…………………………………………………………..3

6. Coverage dependent adsorption characteristics of NMA on the Pt(111) surface……………………………......3

7. Projected density of states for 1/6 coverage………………………………………………………………….4

8. Projected density of states for 1/9 coverage……………………………………………………….............5

9. Projected density of states for 1/16 coverage…………………………………………………………….......6

10. Projected density of states for 1/25 coverage……………………………………………………………….8

11. Projected density of states for 1/36 coverage………………………………………………………..…....10

12. Charge density difference plots……………………………………………………………………….....11

13. Vibrational frequencies of the NMA molecule in gas phase………………………………………………11

14. Tables: Bader Charge Analysis…………………………………………………………………………14

15. Tables: Calculated vibrational frequencies of gas phase NMA ……………………………………………...16

16. Tables: Calculated bond lengths of NMA………………………………………………………………17

17. Temperature-programmed desorption data of NMA……………………………………………………...17

18. X-ray photoelectron spectroscopy data…………………………………………………………………19

19. References……………………………………………………………………………………………...21

1. The band structure of bulk Pt

Our calculated band structure for bulk Pt is in agreement with that in the literature and plotted here just as a reference. It ensures the metallic behavior of the bulk platinum along the K-point path shown in Figure S1.

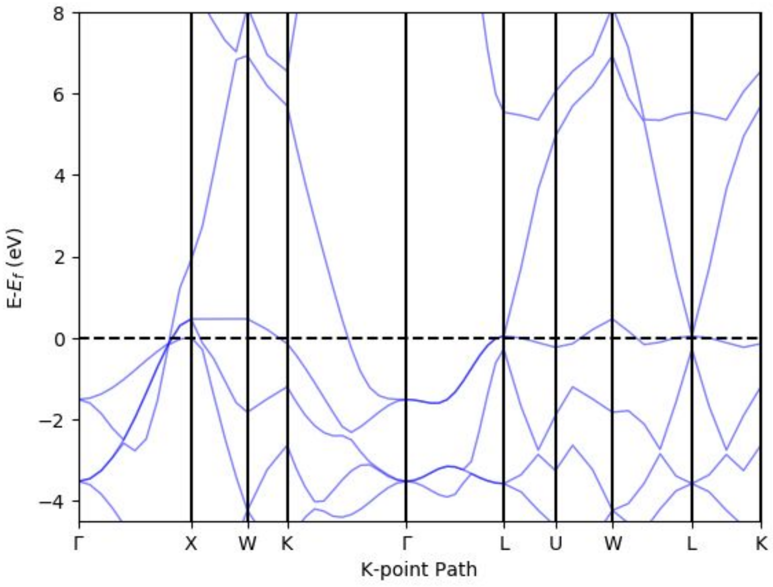

Figure S1: Band structure of bulk Pt.

2. The Pt(111) surface

The binding sites of NMA on the Pt(111) surface considered in this work are shown in Figure S2.

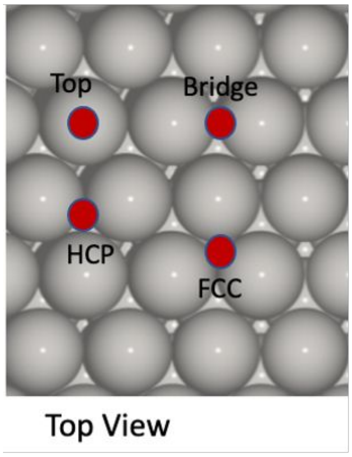

Figure S2: Top view of the fcc (111) plane of Pt surface highlighting the symmetric adsorption sites

3. Projected Density of States for Pt(111) surface and the NMA molecule

The calculated projected density of states of the Pt(111) surface is shown in Figure S3.

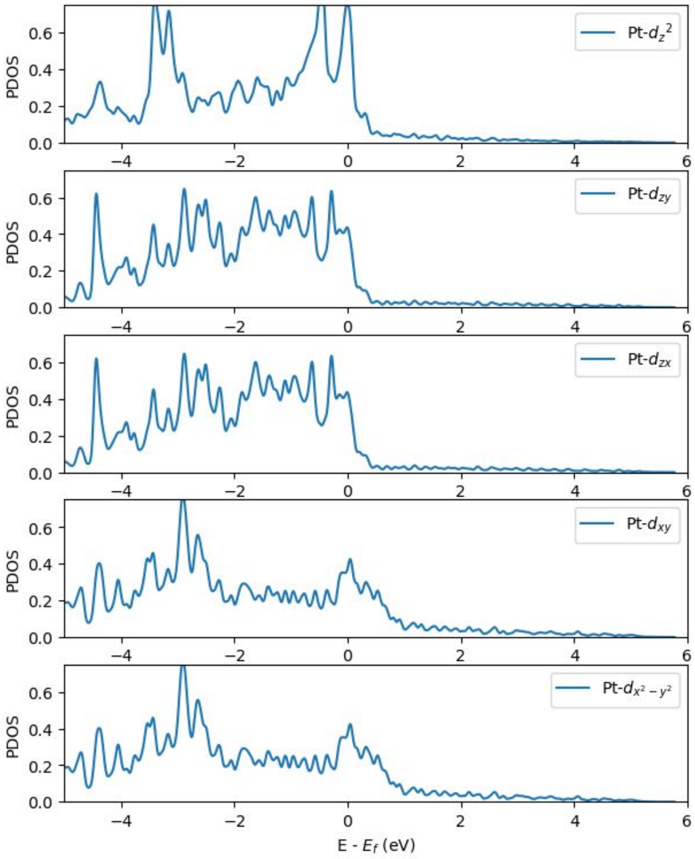

Figure S3: The projected density of states of the Pt(111) surface.

The projected density of states of the nitrogen and phenyl ring carbon atom attached to the nitrogen atom of the NMA molecule in the gas phase are shown in Figure S4(a & b), respectively. The projected density of states of the clean Pt(111) surface and the NMA molecule in gas phase are included here, as they serve as the reference in understanding charge redistribution and hybridization after NMA adsorption, which is further shown in the figures below.

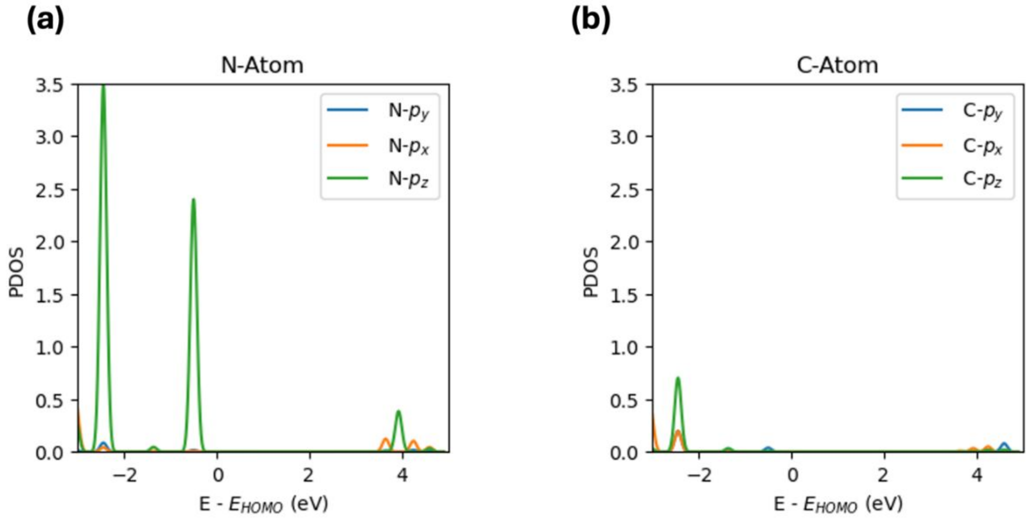

Figure S4: The projected density of states of (a) the nitrogen atom, which shows that p_x and p_z are the frontier orbitals, and (b) the carbon atom, shows p_x and p_y as the frontier orbitals.

4. **Determination of the adsorption site of NMA on the Pt(111) surface**

In Figure S5, each of the three minimum configurations are plotted vs the binding energy (calculated using Equation 1 in the main text. It is found that the top-bridge configuration is the most favorable configuration with respect to adsorption energy.

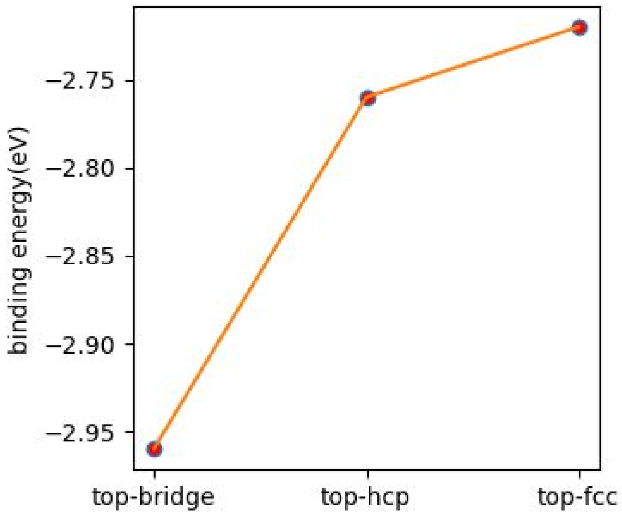

Figure S5: Minimum configuration vs the binding energy plot

The notation top-bridge in the above plot is given as the nitrogen atom of the NMA molecule adsorbs at a top site, and the phenyl ring center is located at the bridge site. Similarly, the notations “top-fcc” and “top-hcp” are assigned.

5. **Binding of the benzene ring and ammonia to Pt(111)**

Our calculations examined the favored adsorption sites for benzene and ammonia on a Pt(111) surface for insights into the adsorption of NMA into Pt(111). Benzene was found to preferentially adsorb at the bridge site[1,2] with a binding energy of -2.95 eV, while ammonia[3] preferentially bound to the top site of a Pt atom on the Pt(111) surface with a binding energy of -0.86 eV. The adsorption behavior of the N-methylaniline (NMA) molecule aligned with that of benzene and ammonia, indicating consistency in the selection of the most stable adsorption sites.

6. **Coverage dependent adsorption characteristics of NMA on the Pt(111) surface**

The height of the nitrogen atom and of the phenyl ring center from the Pt(111) surface vs the coverage are plotted in Figure S6 (a) & (b). The change in the position of the Pt surface atom bond to the nitrogen atom of NMA is plotted in Fig. S6 (c).

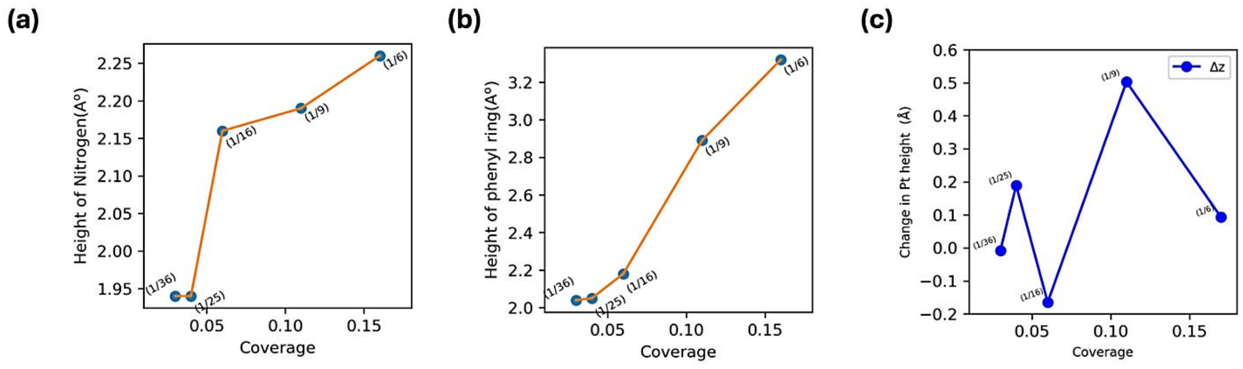

Figure S6: The height (Δz) of (a) the nitrogen atom and (b) the phenyl ring center from the Pt (111) surface (c) the change in position (z-coordinate) of the Pt atom bond to the nitrogen atom for each coverage.

7. Projected density of states for 1/6 coverage

This calculation is for a 2 × 3-unit cell which has six Pt surface atoms, and one molecule adsorbed through the nitrogen atom at the top of the Pt atom. The phenyl ring tilts away from the surface at an incline angle of 33.51°. The projected density of states of the orbitals plotted in Figure S& shows that the p_z orbital of the nitrogen atom is hybridized with the d_{z^2} orbital of the Pt atom that is in contact with it, whereas the carbon atom of the phenyl ring shows no hybridization along the z-axis as they are not in direct contact with the Pt surface.

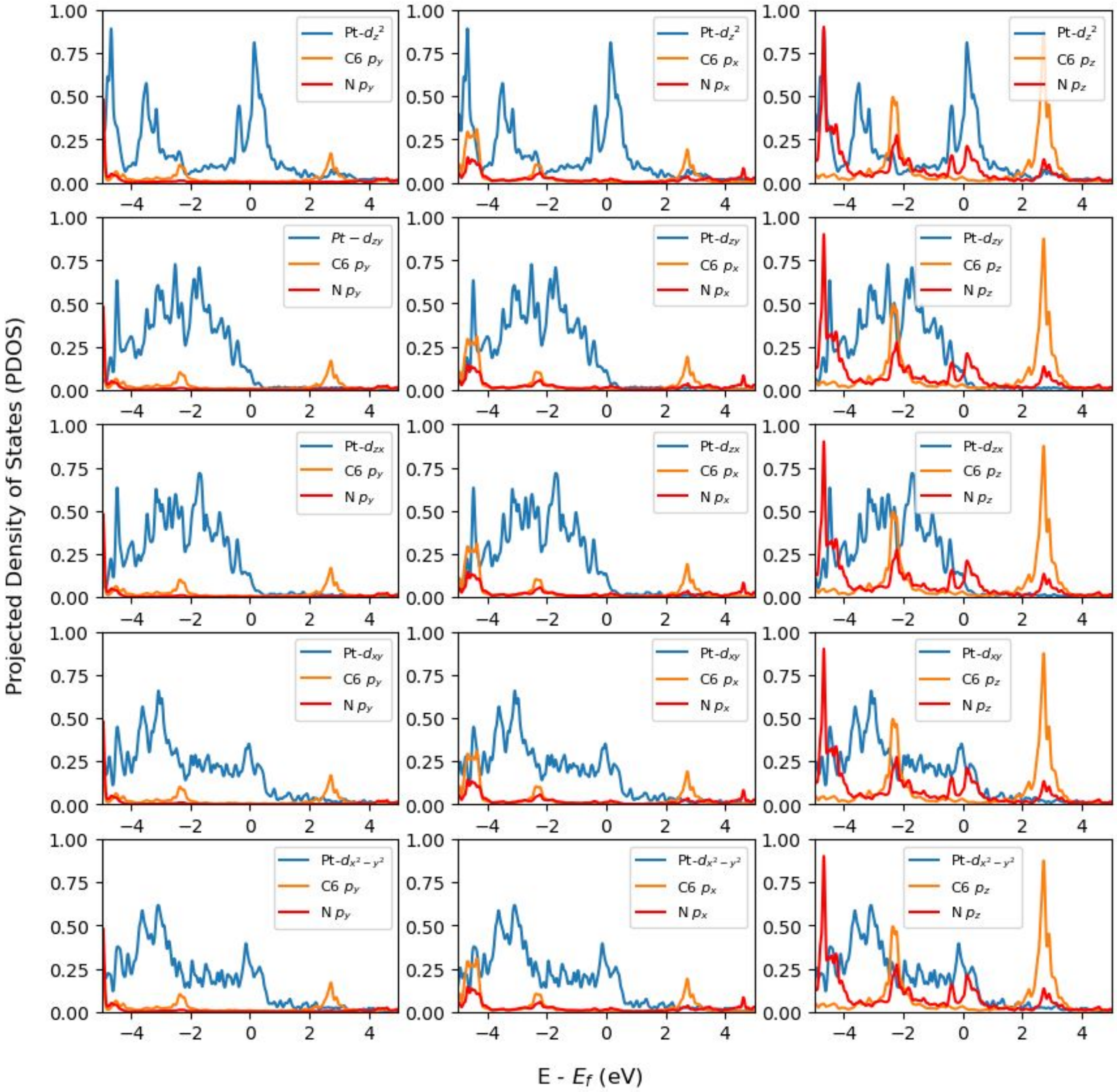

Figure S7: The projected density of states for a 1/6 coverage shows that the Pt d_{z^2} orbital is hybridized with the nitrogen p_z orbital. These subplots represent the partial density of states of the nitrogen atom (p-orbital components) in contact with the surface Pt atom(d-orbital components) and the carbon atom (p-orbital components) of the phenyl ring connected to the nitrogen atom.

8. Projected density of states for 1/9 coverage

The calculation is for a 3 × 3-unit cell which has nine Pt surface atoms, and one molecule adsorbed through the nitrogen atom at the top of the Pt atom. The phenyl ring tilts away from the surface at an incline angle of 12.74°. The projected density of states of the orbitals is plotted and it is seen that the p_z orbital of the nitrogen atom is hybridized with the d_{z^2} orbital of the Pt atom in contact with the nitrogen atom.

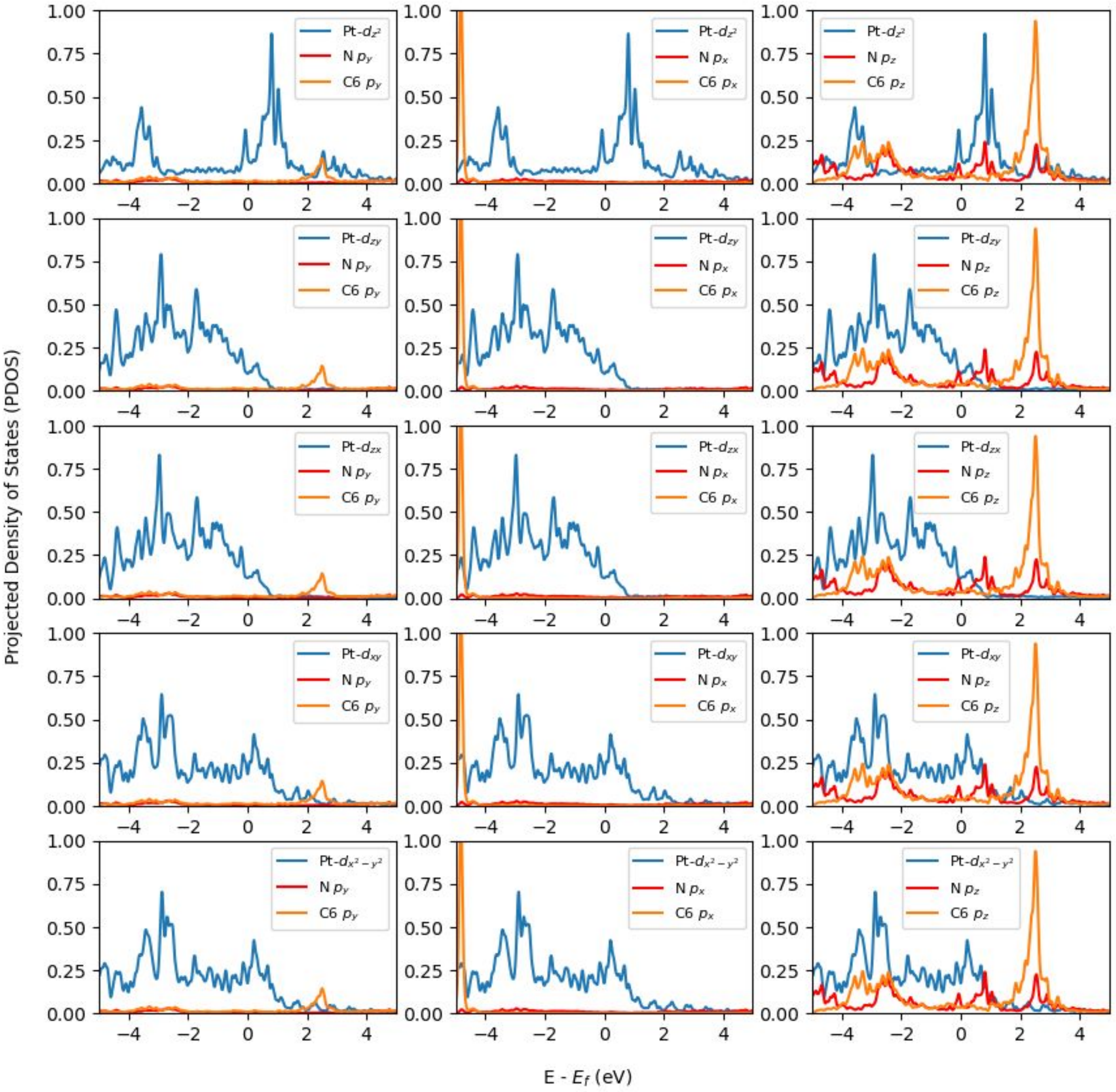

Figure S8: The projected density of states for a 1/9 coverage shows that the Pt d_{z^2} orbital is hybridized with the nitrogen p_z orbital. These subplots represent the partial density of states of the nitrogen atom (p-orbital components) in contact with the surface Pt atom(d-orbital components) and the carbon atom (p-orbital components) of the phenyl ring connected to the nitrogen atom.

9. Projected density of states for 1/16 coverage

This coverage is a 4 × 4-unit cell which has sixteen Pt surface atoms, and one molecule adsorbed through the nitrogen atom at the top of the Pt atom and the phenyl ring from its center at the bridge site. The projected density of states of the orbitals is plotted, and it is seen that the p_z orbital of the nitrogen atom and the carbon atoms of the phenyl ring are hybridized with the d_{z^2} orbital of the Pt atom in contact with the nitrogen and carbon atoms.

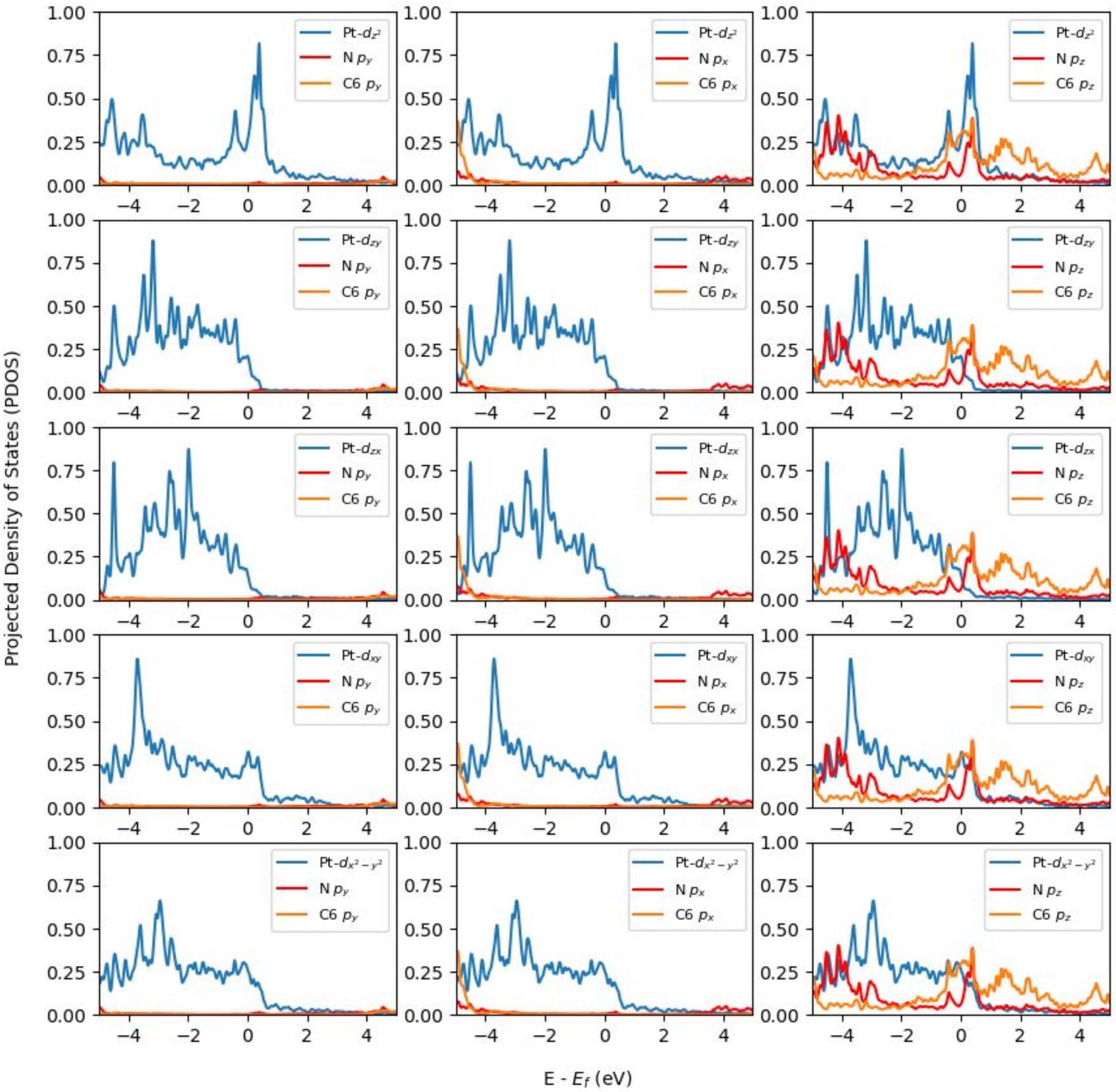

Figure S9-a: These subplots represent the partial density of states of the nitrogen atom (p-orbital components) in contact with the surface Pt atom(d-orbital components) and the carbon atom (p-orbital components) of the phenyl ring connected to the nitrogen atom.

It is shown in Figure S9-a that the nitrogen atom p_z orbital and carbon atom of the phenyl ring (connected to the nitrogen atom) p_z orbital is hybridizing with Pt d_{z^2} orbital. Figure S9-b shows the contribution from other carbon atom of the phenyl ring with the Pt surface atom which is in contact.

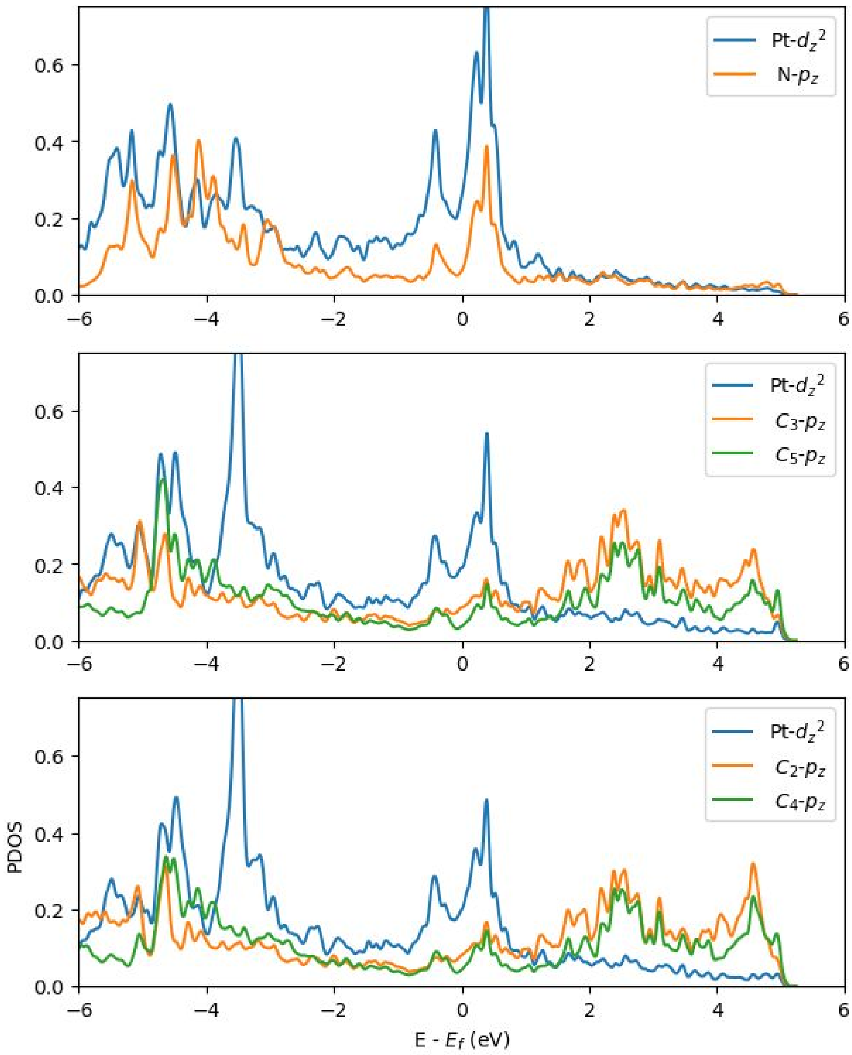

Figure S9-b: The projected density of states for a 1/16 coverage shows that the Pt d_{z^2} orbital is hybridized with the nitrogen p_z orbital and carbon atoms of the phenyl ring p_z orbital

10. Projected density of states for 1/25 coverage:

This coverage is a 5 × 5-unit cell which has twenty-five Pt surface atoms, and one molecule adsorbed through the nitrogen atom at the top of the Pt atom and the phenyl ring from its center of mass at bridge site. The projected density of states of the orbitals is plotted and it is seen that the p_z orbital of the nitrogen atom is hybridized with the d_{z^2} orbital of the Pt atom in contact with the nitrogen atom. The p_z orbital of the carbon atoms of the phenyl ring are hybridized with the d_{z^2} orbital of the Pt atom in contact with carbon atoms.

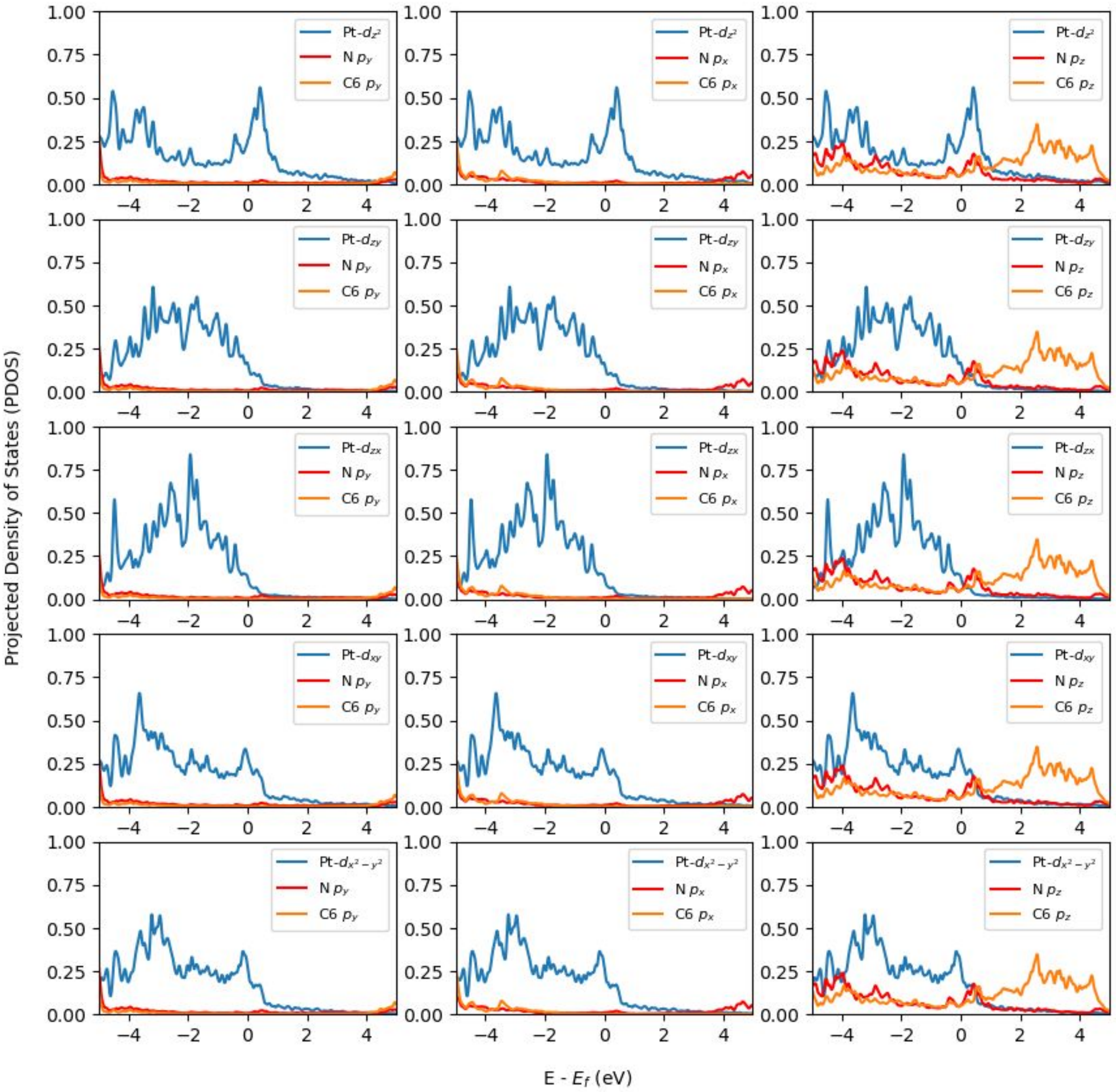

Figure S10-a: These subplots represent the partial density of states of the nitrogen atom (p-orbital components) in contact with the surface Pt atom(d-orbital components) and the carbon atom (p-orbital components) of the phenyl ring connected to the nitrogen atom.

It is shown in Figure S10-a that the nitrogen atom p_z orbital and the carbon atom of the phenyl ring (connected to the nitrogen atom) p_z orbital hybridize with the Pt d_{z^2} orbital. Figure S10-b shows the contribution from another carbon atom of the phenyl ring with the Pt surface atom, which is in contact.

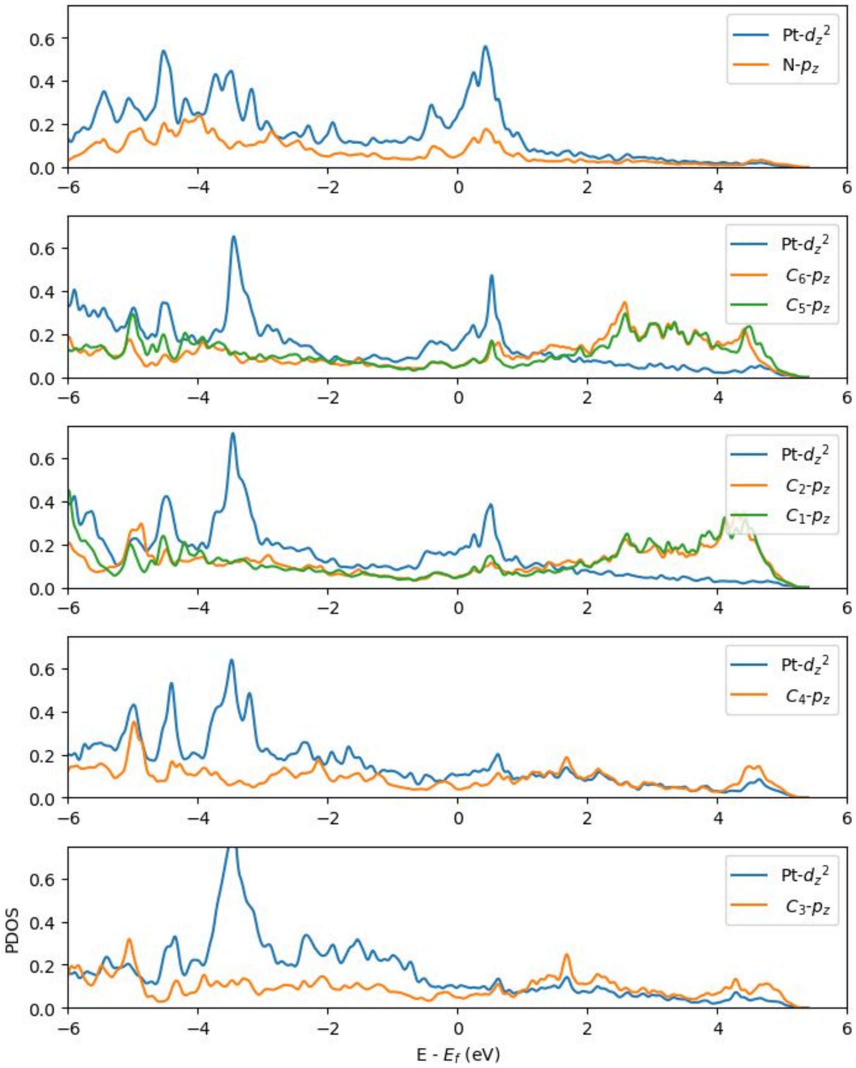

Figure S10-b: The projected density of states for a 1/25 coverage shows that the Pt d_{z^2} orbital is hybridized with the nitrogen p_z orbital and carbon atoms of the phenyl ring p_z orbital

11. Projected density of states for 1/36 coverage

This is a 6 × 6-unit cell which has thirty-six Pt surface atoms, and one molecule adsorbed through the nitrogen atom at the top of the Pt atom and the phenyl ring from its center of mass at the bridge site. The projected density of states of the orbitals is plotted, and it is seen that the p_z orbital of the nitrogen atom is hybridized with the d_{z^2} orbital of the Pt atom in contact with the nitrogen atom. The p_z orbital of the carbon atoms of the phenyl ring are hybridized with the d_{z^2} orbital of the Pt atom in contact with the carbon atoms.

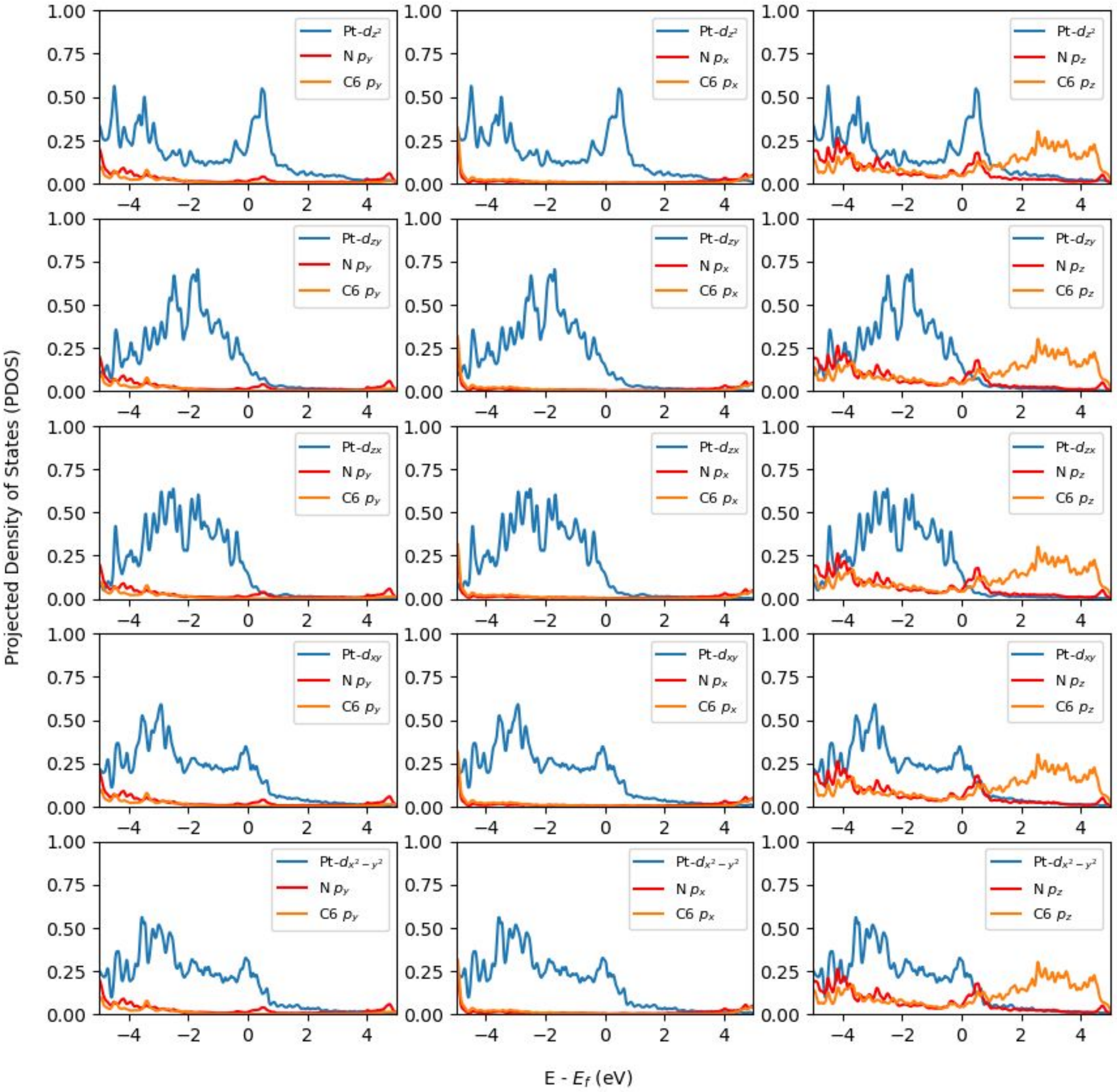

Figure S11: The projected density of states for a 1/36 coverage shows that the Pt d_{z^2} orbital is hybridized with the nitrogen p_z orbital and the carbon atoms of the phenyl ring p_z orbitals

12. Charge density difference plots

The charge density difference, calculated using equation S2, has been plotted using VESTA[4] with iso-value 0.004 e/Å^3, where yellow shows charge accumulation and aqua charge depletion:

$$\Delta\rho = \rho_{Adsorbed\ system} - \rho_{clean\ Pt\ surface} - \rho_{molecule} \qquad Eq.\ (S2)$$

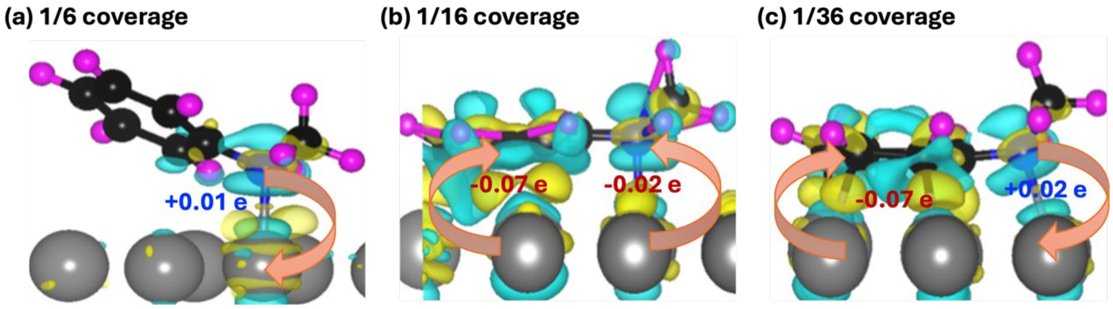

Figure S12: Charge density difference plot: charge sharing among the Pt d_{z^2} orbital with the nitrogen p_z orbital for (a) 1/6 coverage (b) 1/16 coverage (c) 1/36 and charge sharing among Pt d_{z^2} orbital with the carbon atoms of the phenyl ring p_z orbital for (b) 1/16 coverage (c) 1/36 coverage is shown.

13. Vibrational frequencies of the NMA molecule in gas phase

The vibrational modes of the NMA molecule in the gas phase have been calculated using the phonopy code[5] and are in good agreement with previous calculations [6,7] and experimental values summarized in Table 2 in main text. The modes assignments of calculated vibrational frequencies are shown in Figure S13-S20.

The following are the vibrational mode displacement patterns for 1/36 coverage (a), 1/6 coverage (b), and the NMA molecule in the gas phase (c). The number of atoms displaced in the molecule differs between low and high coverages, as illustrated in the images. A comparison of the frequency of the modes for low and high coverage with those of the gas-phase molecule has been conducted, and mode assignments have been analyzed based on frequency and displacement vector directions, as shown in the figures below.

δ(C-H)$_{\text{in-plane}}$

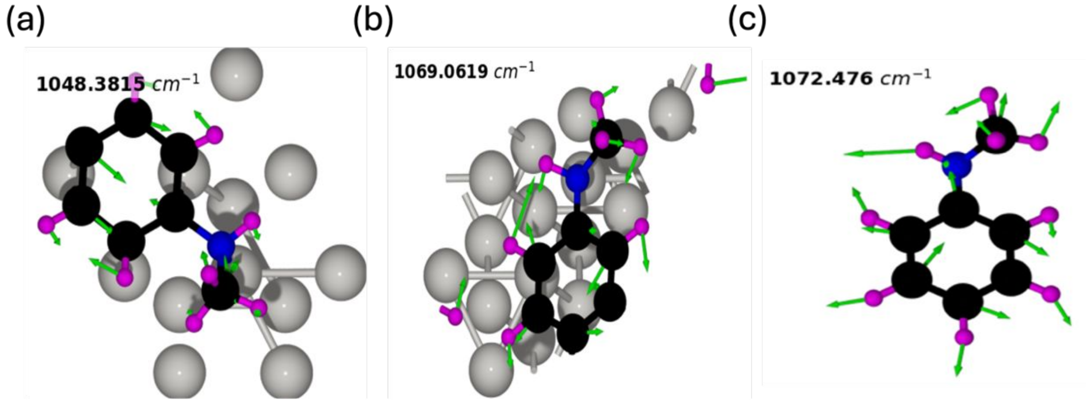

Figure S13: Displacement patterns and frequencies of the δ(C-H)$_{\text{in-plane.}}$ mode of NMA adsorbed on Pt(111) for coverages (a)1/36, (b) 1/6 and (c) the NMA molecule in gas phase

δ(N-H, C-N)

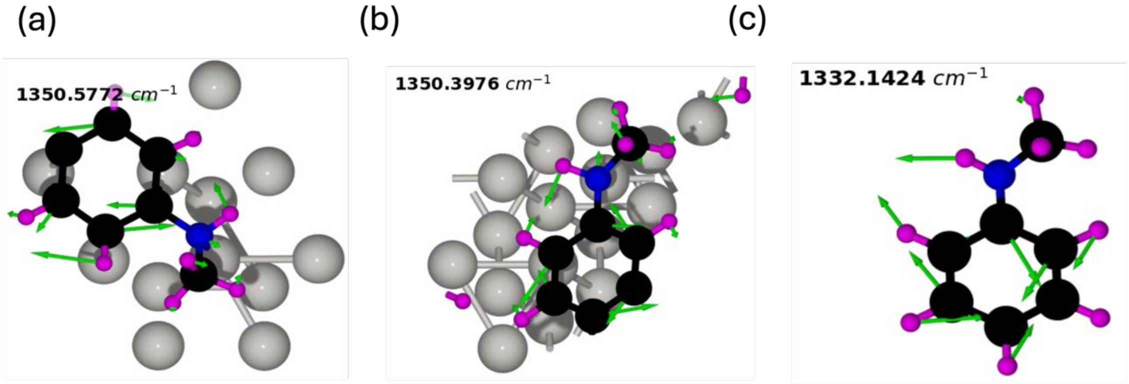

Figure S14: Displacement patterns and frequencies of the δ(N-H, C-N) mode of NMA adsorbed on Pt(111) (a) 1/36 coverage, (b) 1/6 coverage and (c) NMA molecule in gas phase

δ(CH_3)$_{sym.}$

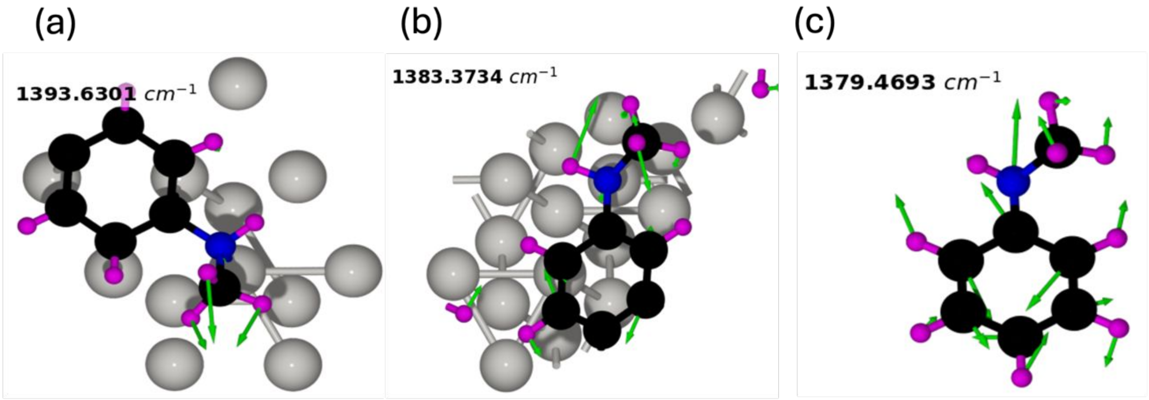

Figure S15: Displacement patterns and frequencies of the δ(CH_3)$_{sym}$ mode of NMA adsorbed on Pt(111) (a) 1/36 coverage, (b) 1/6 coverage and (c) NMA molecule in gas phase

δ(CH_3)$_{asym}$

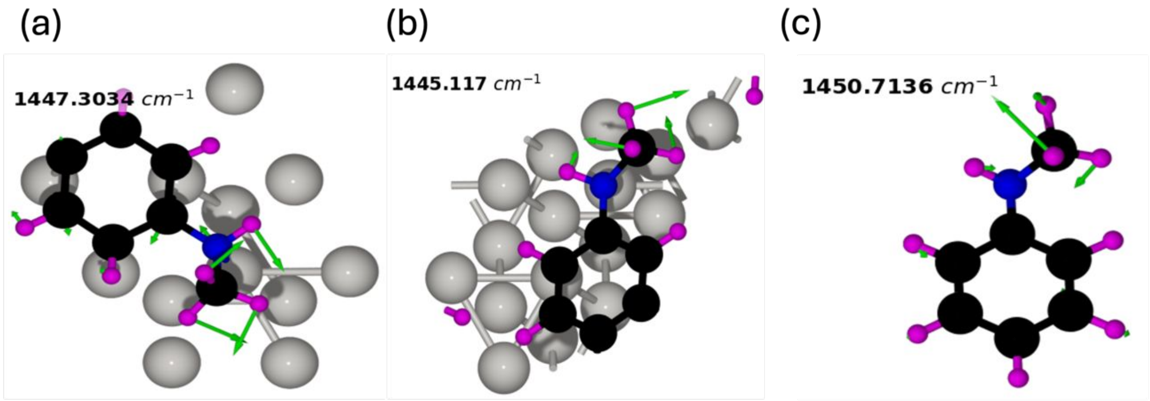

Figure S16: Displacement patterns and frequencies of the δ(CH_3)$_{asym}$ mode of NMA adsorbed on Pt(111) (a) 1/36 coverage, (b) 1/6 coverage and (c) NMA molecule in gas phase

ν(C-C)$_{ring}$

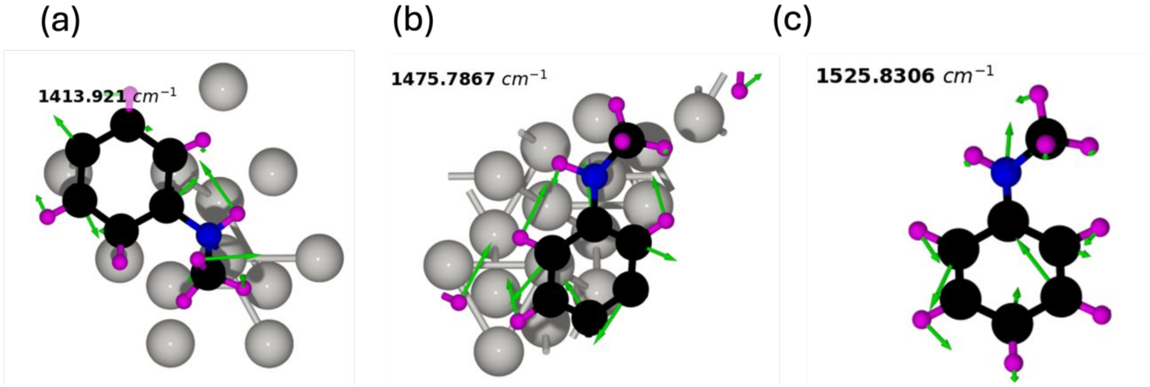

Figure S17: Displacement patterns and frequencies of the ν(C-C)$_{ring}$ mode of NMA adsorbed on Pt(111) (a) 1/36 coverage, (b) 1/6 coverage and (c) NMA molecule in gas phase

ν(CH_3)$_{sym}$

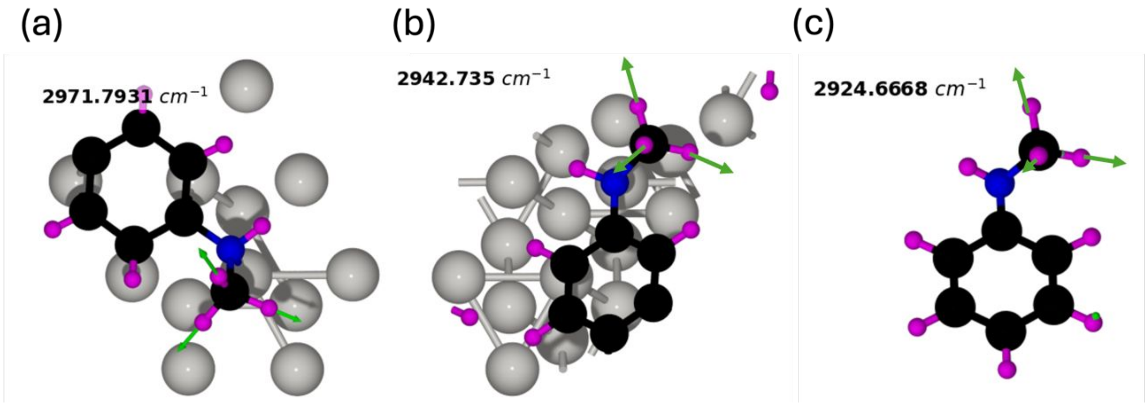

Figure S18: Displacement patterns and frequencies of the ν(CH_3)$_{sym}$ mode of NMA adsorbed on Pt(111) (a) 1/36 coverage, (b) 1/6 coverage and (c) NMA molecule in gas phase

ν(CH_3)$_{asym}$

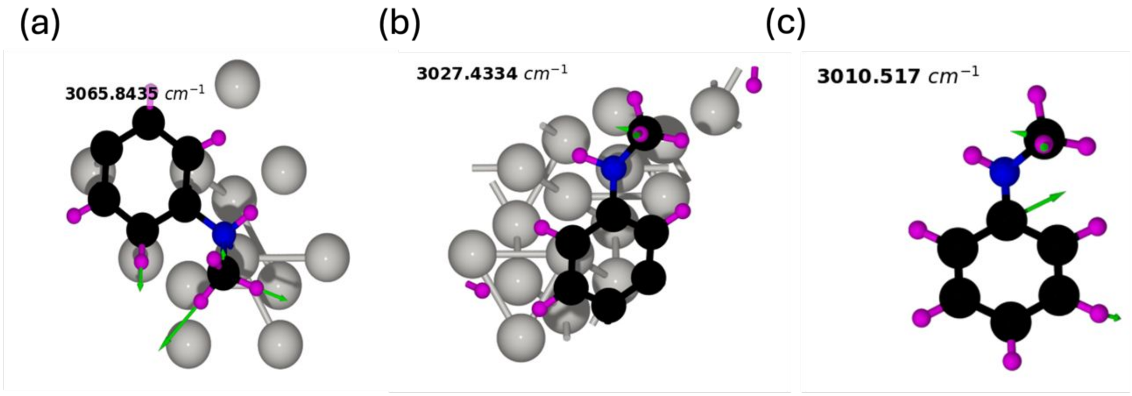

Figure S19: Displacement patterns and frequencies of the ν(CH_3)$_{asym}$ mode of NMA adsorbed on Pt(111) (a) 1/36 coverage, (b) 1/6 coverage and (c) NMA molecule in gas phase

ν(N-H)$_{asym}$

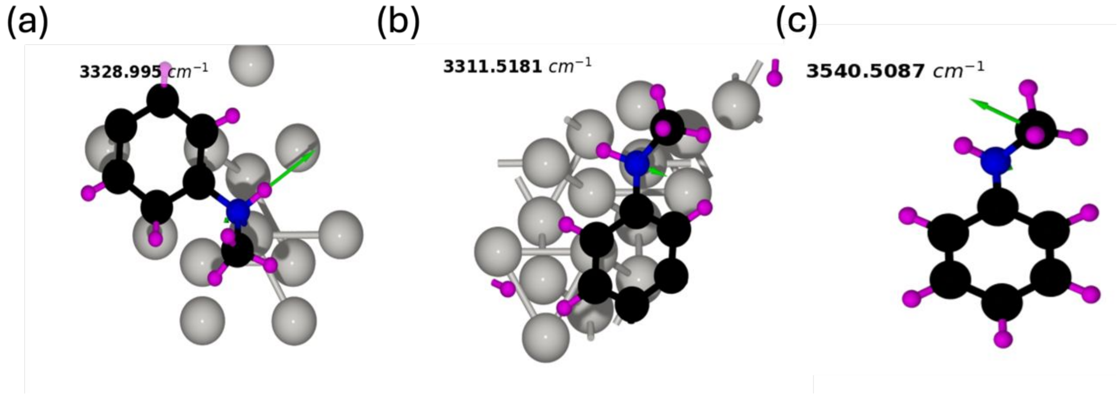

Figure S20: Displacement patterns and frequencies of the ν(N-H)$_{asym}$ mode of NMA adsorbed on Pt(111) (a) 1/36 coverage, (b) 1/6 coverage and (c) NMA molecule in gas phase

14. Tables: Bader Charge Analysis

The charge difference has been calculated using Eq S3.:

$$\Delta q = q_{\text{Adsorbed system}} - q_{\text{molecule(gas phase)}} \quad \text{Eq. (S3)}$$

The charge on each atom is calculated as Z_{value} – charge in ACF.dat file for the respective atom, and the charge difference of each atom is calculated using equation 3 given for the 1/6 and 1/36 coverage as follows:

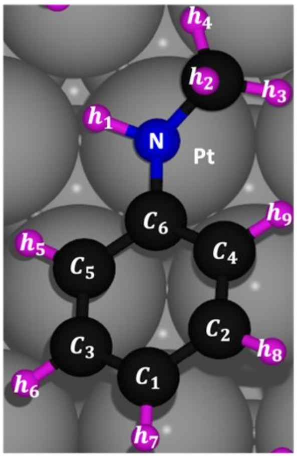

Figure S21: Top view of 1/6 coverage with the labeled atoms where NMA is adsorbed through the nitrogen atom only on the top of the surface Pt atom in contact with it.

Table S1: Bader charge analysis for 1/6 coverage. Label of each atom is shown in the figure above.

Charge on atoms in NMA (gas phase) (e)				Charge on Pt before adsorption (e)		Charge on atoms in NMA after adsorption (e)					
h_1	0.41	C_1	-0.06	Pt	-0.04	h_1	0.44	C_1	-0.04	Pt	-0.07
h_2	0.03	C_2	-0.02			h_2	0.04	C_2	-0.01		
h_3	0.01	C_3	-0.06			h_3	0.04	C_3	-0.05		
h_4	0.05	C_4	-0.07			h_4	0.07	C_4	-0.04		
h_5	0.03	C_5	-0.02			h_5	0.09	C_5	-0.06		
h_6	0.05	C_6	0.30			h_6	0.06	C_6	0.29		
h_7	0.02	C_7	0.24			h_7	0.03	C_7	0.22		
h_8	0.05	N	-1.01			h_8	0.05	N	-1.00		
h_9	0.03					h_9	0.04				

The sum of the charge on the carbon atoms of the phenyl ring after adsorption is 0.14e- and the sum of the charge on the carbon atoms of the phenyl ring before adsorption is 0.21e-, the charge difference using the eq.2 is -0.07e.

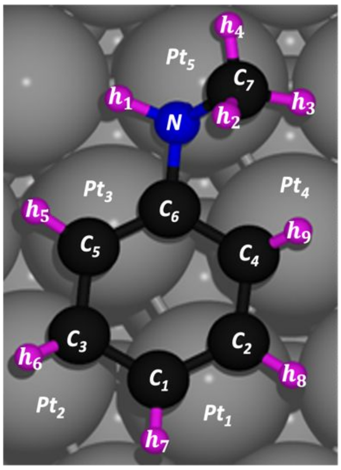

Figure S22: Top view of 1/36 coverage with the labelled atoms where NMA is adsorbed through the nitrogen atom on the top of the surface Pt atom in contact with it and the phenyl ring is adsorbed at the bridge site from the center of mass of the phenyl ring. The phenyl ring is in contact with the four Pt surface atom under it.

Table S2: Bader charge analysis for 1/36 coverage. Label of each atom is shown in the figure above.

Charge on atoms in NMA (gas phase) (e)				Charge on Pt atoms before adsorption (e)		Charge on atoms in NMA after adsorption (e)					
h_1	0.40	C_1	-0.07	Pt_1	-0.05	h_1	0.46	C_1	-0.07	Pt_1	0.04
h_2	0.02	C_2	-0.03	Pt_2	-0.05	h_2	0.05	C_2	-0.06	Pt_2	0.03
h_3	0.04	C_3	-0.07	Pt_3	-0.06	h_3	0.08	C_3	-0.06	Pt_3	0.04
h_4	0.03	C_4	-0.11	Pt_4	-0.06	h_4	0.09	C_4	-0.04	Pt_4	0.02
h_5	0.03	C_5	-0.06	Pt_5	-0.05	h_5	0.08	C_5	-0.06	Pt_5	0.08
h_6	0.07	C_6	0.31			h_6	0.10	C_6	0.23		
h_7	0.04	C_7	0.24			h_7	0.10	C_7	0.20		
h_8	0.03	N	-0.97			h_8	0.11	N	-0.95		
h_9	0.06					h_9	0.08				

15. Tables: Calculated vibrational frequencies of gas phase NMA

Table S3: Comparison of calculated coverage dependent vibrational frequencies with those of gas phase NMA.

**Mode (cm^{-1})**	**2x3**	**3x3**	**4x4**	**5x5**	**6x6**	**Molecule (Gas phase)**
ν(N-H)	3311.5	3325.9	3370.9	3331.6	3328.9	3540.5
$\nu(CH_3)_{asym.}$	3027.4	3042.5	3049.6	3062.9	3065.8	2975.8, 3010
$\nu(CH_3)_{sym.}$	2942.7	2949.8	2959.8	2970.2	2971.7	2924.6
$\nu(C\text{-}C)_{ring}$	1475.7, 1580.7, 1584.7	1466.2, 1561.8, 1566.8	1464.2 1475.9	1452.7	1413.9, 1428.3	1525.8, 1566.6, 1635.02
$\delta(CH_3)_{sym.}$	1383.6	1380.6	1379.8	1393.5	1393.3	1379.4

16. Tables: Calculated bond lengths of NMA

Table S4: Bond lengths: comparison of those in the adsorbed and gas phase NMA

Bond length (Å)	1/6	1/9	1/16	1/25	1/36	gas phase NMA
N-C_6	1.43	1.43	1.39	1.45	1.45	1.38
N-C_7	1.47	1.48	1.47	1.48	1.48	1.44
C_6-C_5	1.40	1.40	1.43	1.44	1.44	1.41
C_6-C_4	1.39	1.40	1.43	1.47	1.47	1.41
C_3-C_5	1.39	1.39	1.43	1.48	1.48	1.39
C_1-C_3	1.39	1.39	1.46	1.47	1.47	1.40
C_2-C_4	1.39	1.40	1.43	1.47	1.47	1.39
C_1-C_2	1.39	1.39	1.46	1.43	1.43	1.39

17. Temperature-programmed desorption data of NMA

According to NIST databank NMA exhibits the following mass fragments: m/z = 107, 106, 79, 78, 77, 51 and 39. NMA desorbs molecularly with mass fragments m/z = 107, 106. The main mass fragment of aniline (m/z = 92) was measured additionally. The mass fragments (m/z = 78, 77) were measured to verify a possible breaking of the C-N bond between amine and ring, which would lead to a desorption of benzene. The following further mass fragments were also measured for checking on possible dissociation of NMA into smaller molecules: H_2 (m/z = 2), CH_4 (m/z = 16), NH_3 (m/z = 17), HCN (m/z = 27), CO/N_2 (m/z = 28), CH_3NH_2 (m/z = 30) and C_2N_2 (m/z = 52). Since no evolution of aniline (m/z = 92), ammonia (m/z = 17) or methane (m/z = 16) were observed during measurements, these mass fragments are not further shown in TPD data. All measured mass fragments are given in the Table S6.

Table S5: List of mass fragments (m/z) and contributors for N-Methylaniline and its decomposition products. The fragmentation pattern was obtained from NIST database and literature.

m/z	contributor	fragment
2	hydrogen	H_2
16	methane	CH_4
17	ammonia	NH_3
27	hydrogen cyanide, NMA	HCN
28	carbon monoxide, nitrogen, NMA	CO, N_2
30	methylamine, ethane	CH_3NH_2
39	benzene, NMA	$C_3H_3^+$
51	benzene, NMA	$C_4H_3^+$
52	benzene, NMA	$C_4H_4^+$ C_2N_2
64	benzene, NMA	$C_5H_4^+$
76	benzene, NMA	$C_6H_4^+$
78	benzene, NMA	C_6H_6
92	aniline	$C_6H_5NH_2$
106	molecular desorption NMA	C_7H_9N

The Figure S23 shows the detected mass fragments in the TPD spectra of an adsorbed multilayer of N-methylaniline at Pt (111). Due to the high number of possible mass fragments, TPD measurements were divided into two parts with m/z <51 and m/z ≥ 51, which were measured separately to avoid temperature shifts due to the dwell times when measured simultaneously

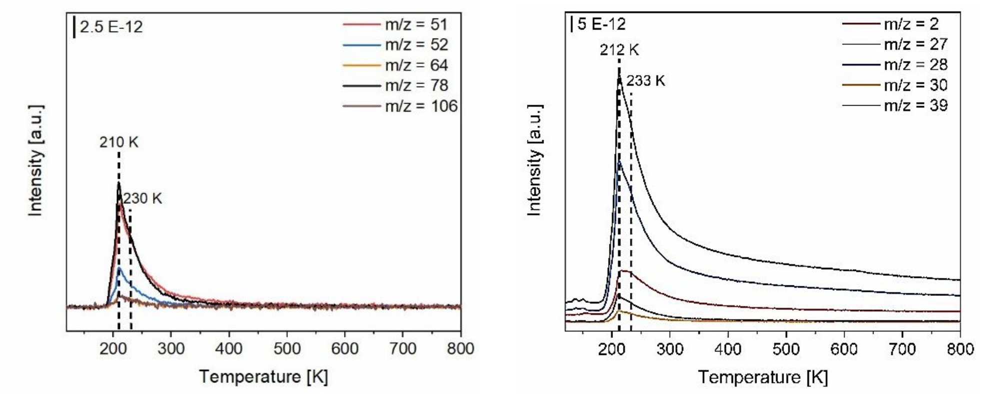

Figure S23: Temperature-programmed desorption spectra (TPD) of an adsorbed multilayer of N-methylaniline (3.5 ML) at Pt (111) with high mass fragments m/z $\geq$ 51 (left) and low mass fragments for m/z $<$ 51 (right). N-methylaniline was dosed through a pinhole doser with a pressure of 1 mbar in the doser compartment while the crystal was cooled down (T < 110 K). TPD data on the left and right were not recorded simultaneously.

Slight temperature shifts of 2 Kelvin between the high and low mass TPD spectra are within the measurement limits. Small deviations could also be possible by slightly different coverages due to the manual opening and closing of the pinhole valve or by measurement inaccuracies of the pressure gauge between the measurements. Both TPD spectra show two temperature features, a main desorption peak at 210 K with a shoulder at 230 K for the mass fragments m/z ≥ 51 and at 212 K and 233 K for mass fragments m/z<51 respectively. The desorption peaks observed here, agree well with results from aniline on Pt (111). For aniline, monolayer desorption was observed at 240 K and multilayer desorption at 200 K[12] .The desorption energy of NMA was determined from the coverage dependent TPD spectra by using the Redhead[13] method. The desorption energy was determined by measuring the temperature of maximum desorption rate of different coverages. Figure S24 shows the graphical analysis using the Redhead method[13].

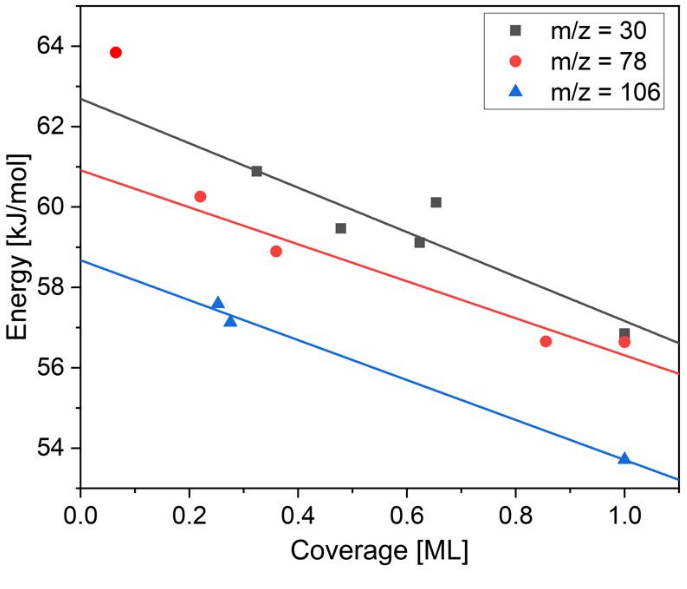

Figure S24: Graphical analysis of varying coverages of N-methylaniline (NMA) at Pt (111) using the Redhead[13] method

The Redhead method was performed for three selected mass fragments (m/z = 106 NMA, m/z = 78 benzene fragment and m/z = 30 methylamine fragment). Using linear regression, the following extrapolated desorption energies are obtained: 58.7 kJ/mol (m/z = 106), 61,1 kJ/mol (m/z = 78), 62.7 kJ/mol (m/z = 30). The deviation in the desorption energy between the different masses lies within the measurement tolerance

18. X-ray photoelectron spectroscopy data

The carbon species of NMA in Figure 12 showed a strong up-shift of 1.6 to 1.9 eV after the adsorption at Pt(111) as mentioned in the paper[6]. This could have several reasons, which shall be discussed below. The binding energy range from 286 up to 288 eV could point to carbon atoms in the neighborhood to oxygen atoms, but O1s spectra (see figure below) do not indicate oxygen species on the surface.

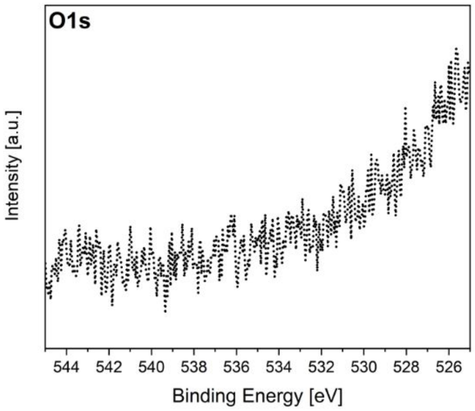

Figure S25: O1s-detailed XP spectra measured after adsorption of a multilayer of N-methylaniline (NMA) at 110 K at Pt (111).

Further reasons for the up-shift as charging effects, coverage-dependent effects, or problems with referencing $Pt_{4f\,7/2}$ at 71.1 eV have been tested, but no significant shift of the C1s signal have been observed, so these effects can be ruled out.

For elucidation of the influence of oxygen on the surface chemistry of NMA, oxygen was dosed through the pinhole doser, after dosing NMA. Figure S26 shows the temperature-dependent XP spectra of NMA and oxygen at Pt(111).

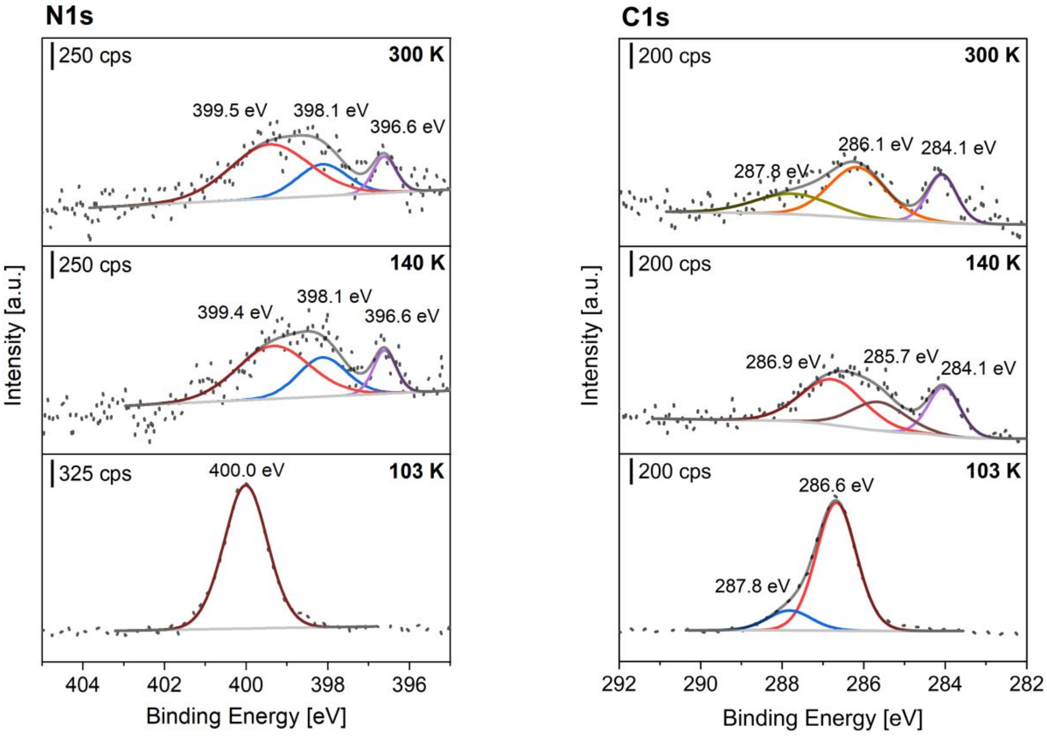

Figure S26: XP spectra of an adsorbed multilayer of N-methylaniline (NMA) co-adsorbed with 50 L oxygen at 103 K at Pt (111) followed by heating to the specified temperatures with the N1s spectra (left) and the C1s spectra (right). The Pt (111) single crystal was heated for 2 min to the elevated temperatures and cooled down afterward.

After co-adsorption of oxygen, the C1s and N1s detailed spectra initially show no difference to pure NMA. However, there are clear differences when heating up the Pt(111) crystal. At 140 K, two species develop in the N1s detailed spectrum that are shifted to lower binding energies. The species can be assigned to a deprotonated imine (398.1 eV)[14,15] and nitrile species (396.6 eV)[16,17]. The intensities decreased significantly at 140 K, indicating also a desorption of the amine like for NMA only at Pt(111). With further heating no significant changes occur in the N1s detailed spectrum at 300 K. The C1s detailed spectrum after co-adsorption of NMA and oxygen show two signals at 286.6 and 287.8 eV in the intensity ratio of 6:1 like NMA only. Heating to 140 K led to the formation of new species at 284.1 and 285.7 eV. The species at 286.9 eV is broadened and up shifted by 0.3 eV. Further heating led to further changes. The species at 285.7 eV and 286.9 eV disappear and a species at 286.1 eV and a shoulder at 287.8 eV occurs. The species at 284.1 eV remain unchanged.

19. References

(1) Ayishabi, P. K.; Lakshmikanth, K. G.; Chatanathodi, R. Chemisorption of Benzene on Pt (1 1 1) Surface: A DFT Study with van Der Waals Interaction. *Chem. Phys. Lett.* **2015**, *637*, 182–188. https://doi.org/10.1016/j.cplett.2015.08.009.

(2) Morin, C.; Simon, D.; Sautet, P. Chemisorption of Benzene on Pt(111), Pd(111), and Rh(111) Metal Surfaces: A Structural and Vibrational Comparison from First Principles. *J. Phys. Chem. B* **2004**, *108* (18), 5653–5665. https://doi.org/10.1021/jp0373503.

(3) Fierro, C. Ammonia Adsorption on a Model Platinum(111) Surface: A Molecular Orbital Approach. *J. Phys. Chem.* **1988**, *92* (15), 4401–4405. https://doi.org/10.1021/j100326a031.

(4) Momma, K.; Izumi, F. VESTA 3 for Three-Dimensional Visualization of Crystal, Volumetric and Morphology Data. *J. Appl. Crystallogr.* **2011**, *44* (6), 1272–1276. https://doi.org/10.1107/S0021889811038970.

(5) Togo, A. First-Principles Phonon Calculations with Phonopy and Phono3py. *J. Phys. Soc. Jpn.* **2023**, *92* (1), 012001. https://doi.org/10.7566/JPSJ.92.012001.

(6) Mudiyanselage, K.; Trenary, M. Adsorption and Thermal Decomposition of N-Methylaniline on Pt(111). *Surf. Sci.* **2009**, *603* (21), 3215–3221. https://doi.org/10.1016/j.susc.2009.09.005.

(7) Yella Reddy, P.; Krishna, T. S.; Gowrisankar, M.; Siva Kumar, K.; Pavan Kumar, C. N. S. S. FTIR Spectra of Pure Components and Their Binary Liquid Components (Binary Mixtures of Formamide with Aniline, N-Methyl Aniline and N,N-Dimethyl Aniline). *Int. J. Ambient Energy* **2022**, *43* (1), 5327–5343. https://doi.org/10.1080/01430750.2021.1945683.

(8) Ansari, A. K.; Verma P. K. Vibrational Spectra of O-Methyl Aniline, m-Methyl Aniline, N-Methyl Aniline & Dimethyl Aniline. *Indian J. Pure Appl. Phys.* **1978**, *16*, 454–458.

(9) Rani, A. U.; Sundaraganesan, N.; Kurt, M.; Cinar, M.; Karabacak, M. FT-IR, FT-Raman, NMR Spectra and DFT Calculations on 4-Chloro-N-Methylaniline. *Spectrochim. Acta. A. Mol. Biomol. Spectrosc.* **2010**, *75* (5), 1523–1529. https://doi.org/10.1016/j.saa.2010.02.010.

(10) Singh, V. B.; Singh, R. N.; Singh, I. S. Vibrational Spectra of Ortho-, Meta- and Para-Chloroanilines. *Spectrochim. Acta* **1966**, *22* (5), 927–933. https://doi.org/10.1016/0371-1951(66)80121-2.

(11) Evans, J. C. The Vibrational Assignments and Configuration of Aniline, Aniline-NHD and Aniline-ND2. *Spectrochim. Acta* **1960**, *16* (4), 428–442. https://doi.org/10.1016/0371-1951(60)80037-9.

(12) Huang, S. X.; Fischer, D. A.; Gland, J. L. Correlation between the Surface Configurations and Hydrogenolysis: Aniline on the Pt(111) Surface. *J. Vac. Sci. Technol. Vac. Surf. Films* **1994**, *12* (4), 2164–2169. https://doi.org/10.1116/1.579107.

(13) Redhead, P. A. Thermal Desorption of Gases. *Vacuum* **1962**, *12* (4), 203–211. https://doi.org/10.1016/0042-207X(62)90978-8.

(14) Bachmann, P.; Schwarz, M.; Steinhauer, J.; Späth, F.; Düll, F.; Bauer, U.; Nascimento Silva, T.; Mohr, S.; Hohner, C.; Scheuermeyer, M.; Wasserscheid, P.; Libuda, J.; Steinrück, H.-P.; Papp, C. Dehydrogenation of the Liquid Organic Hydrogen Carrier System Indole/Indoline/Octahydroindole on Pt(111). *J. Phys. Chem. C* **2018**, *122* (8), 4470–4479. https://doi.org/10.1021/acs.jpcc.7b12625.

(15) Kehrer, M.; Duchoslav, J.; Hinterreiter, A.; Cobet, M.; Mehic, A.; Stehrer, T.; Stifter, D. XPS Investigation on the Reactivity of Surface Imine Groups with TFAA. *Plasma Process. Polym.* **2019**, *16* (4), 1800160. https://doi.org/10.1002/ppap.201800160.

(16) Lindquist, J. M.; Ziegler, J. P.; Hemminger, J. C. Photoelectron Spectroscopy Studies of the Hydrogenation of Cyanogen on Pt(111): Comparison with HCN and Ethylenediamine. *Surf. Sci.* **1989**, *210* (1–2), 27–45. https://doi.org/10.1016/0039-6028(89)90101-5.

(17) Sexton, B. A.; Avery, N. R. Coordination of Acetonitrile (CH3CN) to Platinum (111): Evidence for an H2(C, N) Species. *Surf. Sci.* **1983**, *129* (1), 21–36. https://doi.org/10.1016/0039-6028(83)90092-4.
